# A new set of estimated cardiorespiratory fitness equations are associated with cognitive performance in older adults

**DOI:** 10.1007/s11357-022-00718-w

**Published:** 2023-01-19

**Authors:** Daniel Velázquez-Díaz, Cristina Cadenas-Sanchez, Flor Abril Molina-Guzmán, Jesús Alfredo Sáenz-Carrasco, Javier J. Gonzalez-Rosa, Kirk I. Erickson, Ana Carbonell-Baeza, David Jiménez-Pavón

**Affiliations:** 1 ExPhy Research Group, Department of Physical Education, Faculty of Education Sciences, University of Cadiz, Cádiz, Spain; 2 Biomedical Research and Innovation Institute of Cádiz (INiBICA), Cádiz, Spain; 3 Brain Aging & Cognitive Health Lab, Department of Psychology, University of Pittsburgh, Pittsburgh, PA 15260 USA; 4 MOVE-IT Research Group, Department of Physical Education, Faculty of Education Sciences, University of Cadiz, Cádiz, Spain; 5 PROFITH “PROmoting FITness and Health Through Physical Activity” Research Group, Sport and Health University Research Institute (iMUDS), Department of Physical Education and Sports, Faculty of Sport Sciences, University of Granada, Granada, Spain; 6 Centro de Investigación Biomédica en Red Fisiopatología de la Obesidad y Nutrición (CIBERobn), Instituto de Salud Carlos III, Madrid, Spain; 7 Department of Psychology, Faculty of Education Sciences, University of Cadiz, Cádiz, Spain; 8 AdventHealth Research Institute, Neuroscience Institute, Orlando, FL USA; 9 CIBER of Frailty and Healthy Aging (CIBERFES), Madrid, Spain

**Keywords:** Alzheimer, Cognitive impairment, Physical fitness, Aerobic capacity, Aging

## Abstract

This study aimed to develop new equations to estimate cardiorespiratory fitness specifically for older adults and, secondly, to analyze the associations of cardiorespiratory fitness, both objectively measured and estimated using new equations, with cognitive performance. Ninety-two older adults (41 females, 65–75 years) from baseline data of a randomized controlled trial were analyzed (“ClinicalTrials.gov” Identifier: NCT03923712). Participants completed 4 measurement sessions including (i) physiological and health indicators in a laboratory setting, (ii) field-based fitness tests, (iii) sociodemographic and physical activity questionnaires, and (iv) a battery of neuropsychological tests to evaluate cognitive performance. The main findings were as follows: (i) a set of new equations with good predictive value for estimated cardiorespiratory fitness were developed (74–87%), using different scenarios of complexity and/or equipment requirements, and (ii) higher estimated cardiorespiratory fitness, even using its simplest equation (eCRF =  − 1261.99 + 1.97 × 6 min walking test (m) + 1.12 × bioimpedance basal metabolic rate (kcal/day) + 5.25 × basal heart rate (bpm)), was associated with better cognitive performance evaluated by several neuropsychological tests (i.e., language, cognitive flexibility, fluency, attention, and working memory), similar to using objectively measured cardiorespiratory fitness. In summary, a new set of estimated cardiorespiratory fitness equations have been developed with predictive values ranging from 74 to 87% that could be used based on necessity, availability of equipment, resources, or measurement context. Moreover, similar to objectively measured cardiorespiratory fitness, this measure of estimated cardiorespiratory fitness was positively associated with performance on language, fluency, cognitive flexibility, attention, and working memory, independently of sex, age, and education level.

**Supplementary information:**

The online version contains supplementary material available at 10.1007/s11357-022-00718-w.

## Introduction

Life expectancy of the population around the world is increasing, especially in developed countries; thus, addressing the aging of the population pyramid has become an issue of global concern [1]. During the aging process, there is an increase in the incidence of several diseases, including physical and mental health declines, which have a cascading effect on other health-related problems. Dementia and cognitive impairment are among the most relevant non-communicable diseases or age-related problems [2]. Although there is currently no cure for dementia, non-pharmaceutical strategies such as physical activity or physical exercise interventions are considered essential to prevent disease onset [3]. Particularly, cardiorespiratory fitness (CRF) has been found to be associated with better cognition and lower risk of dementia [4].

CRF is the gold standard for exercise capacity [5], defined as the ability of the circulatory, respiratory, and muscular systems to supply oxygen during sustained physical activity [6]. It is usually assessed as peak oxygen uptake (VO_2peak_) and considered a powerful marker of health [7]. However, to obtain an accurate and precise measurement of CRF, an incremental cardiopulmonary exercise test (CPET) until exhaustion is required [8]. CPET entails certain physical and health risks, especially for older adults, and requires the use of high precision and expensive equipment that hampers its implementation in large-scale studies and clinical settings [5, 9]. In addition, it has been suggested [10] that the main argument frequently used [11], justifying why CRF should not be used in routine clinical practice based on the skills, equipment, and costs associated to CEPT in compared with other assessments [12] is considered as the “old dogma”. However, Kaminsky et al. [13] recently highlighted that commercial metabolic exercise testing units are easy to use providing analysis in real time of respiratory exchange and even built-in electrocardiogram system at lower prices. Because of this, and because of the enormous amount of clinically significant cardiopulmonary data that such units provide, the implementation of CPET in hospitals should now be considered [13]. Therefore, the CRF assessment as “gold standard” using CPET should be used more routinely in clinical practice [14]. However, while CRF is not commonly used in routine clinical practice and other professional settings, one alternative is the use of estimated CRF (eCRF) [15–17]. Several studies [15–17] have previously developed valid equations of eCRF based on large population studies including basic health information in a wide range of ages and aiming to build a simple and cost-effective equation to predict CRF.

Nevertheless, few studies have developed equations to predict CRF in older people. The limited existing evidence has shown that these equations overestimate CRF [18], present high variability in prediction accuracy for those non-maximal, and are not specific for older people, or they were low represented. Therefore, it is of great scientific interest to develop new equations for eCRF that could be easily adopted and have good predictive value using health information available in many healthcare settings.

Previous literature has shown positive associations of objectively measured CRF with brain health in older adults [19–22]. However, although eCRF is frequently used in the clinical setting, few studies have analyzed the association of eCRF with a complete set of cognitive performance tests to determine whether both objectively measured and eCRF show a similar pattern of association with cognitive performance. For example, Boots et al. [23] showed that eCRF was associated with cognitive function; however, this work included people between 20 and 70 years old, and they used an equation that was not specific for older people [17]. Also, Tari et al. [4] concluded that eCRF was an independent risk factor for the incidence of dementia and dementia mortality; however, the association with cognitive performance was not analyzed.

Therefore, the aims of this study were (i) to analyze the accuracy of existing equations in predicting CRF in older adults and to develop new equations to predict CRF (eCRF) in this specific population group and (ii) to analyze the associations of CRF (objectively measured by a laboratory-based test, and estimated by the equations) with a complete set of cognitive performance tests (i.e., screening to cognitive status, language, memory, cognitive flexibility, fluency, inhibition, attention, and working memory).

## Material and methods

### Study participants

The present study used baseline data from the total sample of a randomized controlled trial (RCT registered in “ClinicalTrials.gov,” Identifier: NCT03923712). Ninety-two older adults 41 women were recruited in 13 public health care centers from Cádiz. The inclusion criteria were as follows: between 65 and 75 years, being able to speak, understand and write Spanish properly, not suffering any disease/injury that prohibits engagement in physical activity, and not engaging in supervised physical activity for more than 20 min/day. Exclusion criteria were as follows: suffering from an acute or terminal disease, chronic depression and/or unstable cardiovascular disease, and having a medical history of head injury with loss of consciousness or ictus.

Participants were informed about the study procedures, potential risks, and benefits. If they met the inclusion criteria and agreed to participate, they signed the informed and photo/video consent form. This study was approved by the Human Ethics and Research Committee of the research in Cádiz and the Andalusian Coordinating Committee on Biomedical Research Ethics (codes: 0667-M1-17 and 04/2018, respectively) and conducted in accordance with the Declaration of Helsinki.

### Measurements

There were four different assessment days for the measurements of the following: (i) physiological and health indicators in a laboratory setting, (ii) field-based fitness tests, (iii) sociodemographic and physical activity questionnaires, and (iv) a neuropsychological evaluation.

### Physiological and health indicators in the laboratory setting

Participants performed a set of laboratory tests and were instructed to follow several considerations previous to the evaluation. These standardized considerations included refraining for the 24 h prior to the assessment from (i) strenuous physical exercise, (ii) intake of alcohol, caffeine and energetic drinks, and (iii) to control hydration status during the previous week. In addition, on the evaluation day, participants were instructed to fast for at least 4 h before the scheduled session [24–26].

#### Body composition

Body weight, fat mass, fat-free mass, and estimated basal metabolic ratio (eBMR) were obtained using a multifrequency bioimpedance (TANITA-MC780MA) [24, 25, 27]. Height was measured by a stature-measuring instrument (SECA 225, Hamburg, Germany) [28]. Body mass index (BMI) was calculated (weight (kg)/height^2^ (m)). Waist circumference was assessed with a tape (Lufkin W606PM) following the ISAK protocol [29].

#### Pulmonary capacity

A forced spirometry in a standing position was performed using Jaeger MasterScreen CPX_®_ (CareFusion, San Diego, USA). After several cycles of normal breathing, the participants were instructed to inspire as much air as possible, with a pause of less than 1 s, to later expire the air as quickly as possible being prolonged until the participants were unable to expire more air or evaluator indication. Forced expiratory volume in 1 s (FEV_1_), forced vital capacity (FVC), peak expiratory flow, and FEV_1_/FVC values were registered and calculated according to the SEPAR normative [30]. The test was repeated after resting for 2–3 min until at least two attempts of the test were considered acceptable.

#### Basal metabolic rate (BMR)

BMR was assessed by indirect calorimetry using a gas analyzer of open circuit (Jaeger MasterScreen CPX_®_; CareFusion, San Diego, USA) according to the established criteria for measuring BMR for 10 min on a bed [26]. Heart rate (HR) was measured with Polar Team System 2 Pro (Polar Electro Oy, Kempele, Finland), and the lowest HR was recorded as basal HR [31].

#### Cardiopulmonary exercise test (CPET)

An incremental CPET until exhaustion using the modified Bruce protocol to determine objectively CRF was performed on a treadmill (Lode Valiant, Groningen, Netherlands) [32]. Participants began walking at 2.7 km/h at 0% inclination grade, and every 2 min, the speed or/and inclination were increased according to the protocol. Respiratory exchange ratio (RER), VO_2_, and VCO_2_ consumption were measured breath by breath using indirect calorimetry through a gas analyzer of open circuit, Jaeger MasterScreen CPX® (CareFusion, San Diego, USA) daily calibrated for each test. In the middle of each step, a rating of perceived exertion (RPE) was asked using the 10-point Borg [33]. HR was continuously monitored. The CPET was considered maximal if the test met at least three of the following criteria: (i) RER ≥ 1.05, (ii) a plateau in VO_2_ achieved in the last three intervals of 10 s (< 2 ml·kg^−1^·min^−1^), (iii) subjective volitional exhaustion, (iv) HR ≥ 85% theoretical maximum HR (HR_max_), and (v) RPE ≥ 7 [33–36]. VO_2peak_ was established as the highest observed value of oxygen consumption obtained in the last three intervals of 10 s of the CPET; this parameter was used in the analyses as objectively measured CRF.

### Field-based fitness tests

Two tests of the senior fitness test battery [37] and handgrip test were applied to assess CRF and muscle strength.

#### Aerobic endurance

Aerobic endurance was assessed by the 6-min walking test which consists of walking as fast as possible (without running) between two cones 30 m apart. The test was performed only once at the end of the evaluation session, and the total of walked meters during 6 min was registered and used for analyses.

#### Muscle strength

A handgrip test was performed to assess upper body strength using a digital dynamometer (TKK 5101 Grip-D, Tokyo, Japan) [38]. To adjust a correct grip, the dynamometer was fixed at 5.5 cm size for males, and for females, the optimal grip was calculated according to the hand size [38]. Participants had to maintain the standard bipedal position during the entire assessment, with the elbow in a complete extension. Two attempts were performed with each hand, and the best value of each hand was averaged and used for analyses. The scores were recorded in kilograms. To assess lower-body strength, we used the chair stand test. Participants had to stand up and sit down as fast as possible for 30 s with arms folded across the chest. The test was performed only once, and the total number of repetitions was recorded and used for analyses.

### Sociodemographic and physical activity questionnaires

A modified sociodemographic questionnaire based on the Spanish national health survey [39] was used to collect information about several dimensions such as marital status, educational level, education years, household income, smoking, medication, pathologies, and alcohol or tobacco consumption. Moreover, the Global Physical Activity Questionnaire (GPAQ), which is a valid and self-reported questionnaire to assess physical activity in three different domains (work, transport, and leisure time), was applied [40]. Participants were categorized as reaching at least 150 min a week of physical activity or not in their leisure and work time.

### Neuropsychological evaluation

A comprehensive neuropsychological test battery measured cognitive performance including eight internationally well-known and gold-standard and validated instruments for older adults [41–48]. The tests assess cognitive impairment, learning and verbal episodic memory, visuoconstructive and visuospatial skills, verbal and semantic fluency, visual confrontation naming, cognitive flexibility, attention, and inhibition. All the neuropsychological assessments were administered in a single session that lasted no longer than 80 min. Briefly, The neuropsychological assessment consisted of the following:

- The Mini-Mental State Examination (MMSE) is a valid test widely used to evaluate cognitive status [49], with a total score ranging from 0 to 30, where the highest score is the best performance.
- The Boston Naming Test (BNT) is a valid and widely used test for assessing language dimension [43]. Particularly, we used the short version of the BNT 15 items. Participants were shown simple and rare line drawings of objects and asked to name them orally. Total punctuation was the sum of the total correct responses, where the higher the correct responses the better the cognitive performance.
- The Clock Drawing Test (CDT) is a valid cognitive test used for dementia screening [42]. This test consisted of drawing a clock with the numbers on the circle, showing the clock hands at a specific time (11:10). The total score is calculated in a range of 0 to 10, where the highest score reflected the best performance [50].
- The Rey Auditory Verbal Learning Test (RAVLT) is a valid test to assess learning and verbal episodic memory [44]. A higher score reflected a greater number of words learned over 5 verbal presentations of 15 words.
- The Trail Making Test (TMT) is a valid test used to assess cognitive flexibility and alternating attention [45] and consists of two parts (A and B). The completion time of both parts was registered in seconds, and the interference (time record of part B–time record of part A) was the continuous variable used for analyses (the lower duration, the better the performance).
- The Controlled Oral Word Association Test (COWAT) is a valid instrument for assessing verbal and semantic fluency [46]. This test consisted of the spontaneous production of words beginning with a designated letter and a topic determined (P, M, and R for the Spanish version) within a minute for each letter. Total punctuation was calculated by summing all items independently for each letter; thus, the higher number of words given, the better the performance.
- The Stroop Color and Word Test (Stroop) is a valid and widely applied test for examining cognitive flexibility, selective attention, and cognitive inhibition [47]. This test was divided into three conditions containing 100 words each, and the time was limited to 45 s per condition. The total number of correct words for each condition was registered indicating that the higher number of correct words, the better the performance.
- The Digit Span task is a subtest of the Wechsler Adult Intelligence Scale (WAIS)-III scale [48]. Firstly, this test consisted of a direct digit-sequencing (Digit span forward), and secondly, the digits are presented in reverse order (Digit span backward) to asses attention and working memory, respectively. Each correct item provides one point, and the total is computed ranging from 0 to 16 in both parts, where the higher scores indicate better performance.

Raw scores of each test were transformed into *z*-scores to generate an overall z-score as the mean of the nine standardized *z*-scores for the neuropsychological tests (standardized value = (mean value)/SD). To do so, the TMT score was reverted (multiplying by − 1) in order to present all neuropsychological tests following the same direction (the higher the result, the better the performance).

### Statistical analyses

The normality of the variables was checked using both graphical and statistical procedures. To test sex for differences, a *t*-test was applied. To analyze the accuracy of existing equations in predicting CRF in older adults, we apply each previous equation using the current population data of older adults of this study. After this, the delta (∆) was calculated by objectively measured CRF by indirect calorimetry—eCRF value with each equation, and linear regression analyses were also applied to obtain the *r*^2^ for each equation using the data of our study sample. Then, to develop equations for estimating CRF in older adults, a statistical approach using stata was applied. Briefly, the *maxvar* subcommand was used to select the main predictor variables, and then, the *allset* regression command was applied to propose three new CRF prediction models. Prediction model 1 included basic variables such as body composition, meeting physical activity recommendations (yes or no), field tests, and basal parameters, which was called “The Basic Equation.” Then, spirometry parameters were added in prediction model 2 (named as “The Extended Equation”). In prediction model 3 (named as “The Maximal Equation”), the variables registered during CPET were included. Then, the five best equations of each prediction model were selected between a total of 65,535 possible regressions, automatically executed by the software, using as criteria the coefficients of Mallow’s Cp, *r*^2^, adjusted *r*^2^, Akaike’s information criterion, and the Bayesian information criterion. Finally, 1 out of 5 prediction equations from the three prediction models was suggested to be used in further analyses. Additionally, Bland‐Altman plots to display systematic and random error of the newly developed equations were performed.

Then, multiple linear regression analyses were applied to analyze the association of objectively measured CRF and eCRF with cognitive performance. The unadjusted model (model 1) and the adjusted model (model 2) including sex, age, BMI, and/or education level as covariates were used for the regression analysis. This model 2 was based on scientific criteria, where both the individual association of potential confounders and its modifying effects over the coefficient (> 10%) were analyzed. Moreover, the interaction was also verified for the included confounders by generating virtual dummy variables in STATA code (independent × confounder) and checking its significance. This process of building the adjusted model was done for each independent variable (CRF and eCRF). The full process was performed for all neuropsychological tests as dependent variables and an overall *z*-score. This *z*-score of cognitive performance was calculated as the mean of the nine standardized scores for the neuropsychological tests (standardized value = (mean value)/SD). To do so, the TMT score was reverted (multiplying by − 1) in order to present all neuropsychological tests following the same direction (the higher the result, the better the performance).

Finally, additional sensitivity analyses were performed only for those participants achieving maximal criteria in CPET or using relative CRF instead of absolute CRF. Moreover, the normality of the residuals and the collinearity of the regression models were verified (command.*vif*, for STATA).

All analyses were performed using the STATA software for Windows version 13.0. The level of significance was set at *p* < 0.05.

## Results

### Participants

**Fig. 1:**
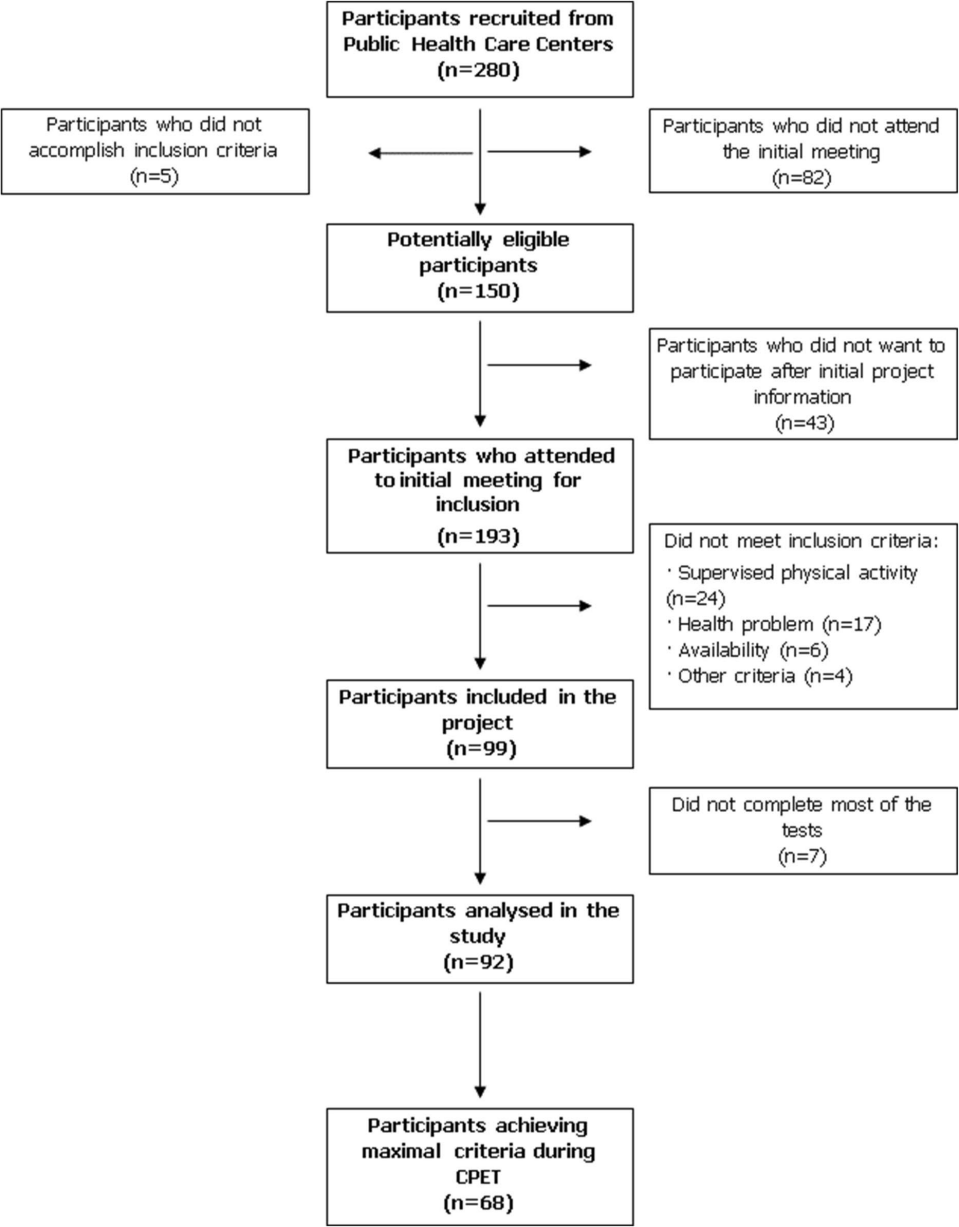
Flow chart of participants recruitment. CPET, cardiopulmonary exercise test

Figure 1 shows the flow chart of participant recruitment. Two hundred and eighty people were called to informative meetings to describe the characteristics of the project. Of these, ninety-two participants (41 females) were finally included in the present study (Fig. 1).

### Descriptive characteristics

Descriptive characteristics of the study participants are shown in Table 1, and additional descriptive information can be found in the supplementary material (Supplementary Table S1). There was no difference in age between sexes, but significant differences were found for the anthropometric variables (all *p* < 0.050; except for BMI). Moreover, males reported higher values for BMR (*p* < 0.001), spirometry (all *p* < 0.001; except for FEV_1_/FCV), and physical fitness measures (all *p* < 0.001) than females. Finally, sex differences for two neuropsychological tests (BNT and COWAT) were found, with males having higher scores (*p* < 0.001 and *p* = 0.011, respectively). There was a significant difference between sexes for the overall *z*-score of cognitive performance (*p* = 0.031).

**Table 1:** Descriptive characteristics of the study participants

Variable		Total sample *n* = 92				Male *n* = 51			Female *n* = 41		*p*-value
Physical characteristics											
Age (years)		68.9± 2.9			69.1±2.9			68.7± 2.9		0.522	
Weight (kg)		74.5±13.8			80.4±13.5			67.0 ± 9.9		** < 0.001**	
Height (cm)		161.1±9.6			166.9±7.4			153.7±6.6		** < 0.001**	
Body mass index (kg·m^−2^)		28.7 ± 4.5			28.9 ±4.4			28.4 ±4.7		0.661	
Resting metabolic parameters											
Indirect calorimetry BMR (kcal·day^−1^)		1648±338	1827±307					1425±225			** < 0.001**
Bioimpedance estimated BMR (kcal·day^−1^)		1511±284	1707±218					1261±107			** < 0.001**
Spirometry*											
FEV_1_ (l)		2.45±0.60	2.70±0.59					2.14±0.69			** < 0.001**
FVC (l)		3.66±1.13	4.24±1.09					2.97±0.73			** < 0.001**
FEV_1_/FVC (%)		72.1±14.1	69.7±15.0					75.1±12.5			0.065
Physical fitness measures											
* Cardiopulmonary exercise test*											
Absolute cardiorespiratory fitness (ml min^−1^)	1853±482			2141±403			1478±273				** < 0.001**
Relative cardiorespiratory fitness (ml·kg^−1^·min^−1^)	25.0±5.2			27.1±5.0			22.3±4.3				** < 0.001**
*Physical fitness field tests*											
6 min walking (m)	554.2±83.1			582.5±88.3			519.9±61.6				** < 0.001**
Handgrip (kg)	29.1±8.9			35.5±6.6			21.2±3.4				** < 0.001**
Chair stand (rep)*	11.2±2.5			11.9±2.7			10.2±1.9				** < 0.001**
Sociodemographic characteristics											
Marital status (S/M/W/D)	(5/79/10/6)			(0/84/8/8)			(10/73/12/5)				
Household incomes (High/medium/low)**	(15/40/45)			(14/56/30)			(10/38/32)				
Education level (years)	10.9±9.0			11.9±10.2			9.7±7.2				0.249
Smoking (yes/no)	(34/66)			(29/71)			(39/61)				
Cognitive performance											
Mini mental state examination (total score, 0–30)	27.8±2.2			27.9±1.8			27.6±2.6				0.467
Boston naming (total score, 0–15)	10.8±2.3			11.7±2.0			9.8±2.1				** < 0.001**
Clock drawing (total score, 0–10)	7.4±1.9			7.6±2.0			7.2±1.9				0.332
Rey auditory verbal learning (total score, 0–15)	12.5±2.7			12.1±3.1			13.1±1.9				0.060
Trail making (s)	101.4±92.3			94.7±90.0			109.4±95.5				0.444
COWAT (total score, words in 60 s)	35.1±13.4			38.3±14.0			31.5±11.7				**0.011**
Stroop (total score)*	4.4±7.3			4.1±7.6			4.8±7.0				0.640
WAIS Forward (total score, 0–16)*	8.0±2.1			8.1±1.9			7.7±2.3				0.471
WAIS Backward (total score, 0–14)*	4.9±2.5			5.25±2.5			4.4±2.3				0.156
* z*-score (score)	0.01±0.7			0.13±0.7			-0.16±0.6				**0.031**

Values are presented as mean ± SD. *T*-test square statistics was applied. Statistically significant differences between sexes are highlighted in bold

*BMR*, basal metabolic rate; *COWAT*, controlled oral word association test; *D*, divorced; *FEV1*, forced expiratory volume in 1 s; *FVC*, forced vital capacity; *M*, married; *S*, single; *SD*, standard deviation; *WAIS*, Wechsler Adult Intelligence Scale; *W*, widowed

^*^Subsample of 90 participants in all cases, except for WAIS, 81

^**^Household incomes higher than 5000€/month, high; between 3,000€ and 1,500€, medium; lower than 1,500€, low

### Prediction equations for cardiorespiratory fitness

#### Examining the accuracy of existing equations in predicting CRF

eCRF equation characteristics of the previously published equations can be found in Supplementary Table S2. No equation obtained a good prediction ratio after applying to our study sample (Supplementary Table S2, all *r*^2^ < 0.45). The difference between eCRF by previous equations and the objectively measured CRF ranged from *-16.36* to *16.48* ml/kg/min (Supplementary Table S2). Indeed, only three equations present a delta difference of ~  ± 1 ml/kg/min. Moreover, using the same predictors as previous equations, we observed that they did not achieve a high prediction ratio for CRF (Supplementary Table S3, all *r*^2^ < 0.50).

#### Developing new equations to predict CRF

Table 2 shows the main predictors of CRF level for the overall sample. There were significant associations for most of the predictors with absolute CRF levels (all *p* < 0.050). No association was observed for age, physical activity, and RPE. In sensitivity analysis, a similar trend was observed with those participants achieving maximal criteria in CPET and, when the analyses were repeated with relative CRF, instead of absolute, as a dependent variable (data not shown).

**Table 2:** Independent predictors of CRF (ml/min)

	*B*	*β*	*r*^2^	*p* value
Sex (male/female)	− 667.352	− 0.692	0.48	** < 0.001**
Age (years)	− 29.039	− 0.181	0.03	0.084
Basal metabolic rate (kcal·day^-1^)	0.834	0.588	0.35	** < 0.001**
6-min walking (m)	2.700	0.465	0.22	** < 0.001**
Weight (kg)	21.198	0.606	0.36	** < 0.001**
Waist circumference (cm)	16.259	0.393	0.15	** < 0.001**
HR basal (bpm)	11.050	0.207	0.04	**0.048**
Handgrip (kg)	20.050	0.711	0.51	** < 0.001**
Chair stand (rep)	107.63	0.492	0.24	** < 0.001**
Arm curl (rep)	18.391	0.270	0.07	**0.010**
FEV1 (l)	323.96	0.463	0.21	** < 0.001**
Smoking (yes, no)	− 93.73	− 0.185	0.18	** < 0.001**
Meeting PAr (yes, no)	235.45	0.222	0.05	0.055
Maximum HR (bpm)	12.456	0.503	0.25	** < 0.001**
Time to exhaustion (min)	66.334	0.526	0.28	** < 0.001**
RPE max (1–10)	34.643	0.166	0.03	0.112

Statistically significant values are shown in bold

*B*, regression coefficient; *β*, standardized coefficient; *FEV*_*1*_, forced expiratory volume in 1 s; *HR*, heart rate; *PAr*, physical activity recommendations; *r*^2^, adjusted *R*-squared; *RPE*, rating perceived exertion

Table 3 shows the fifteen best prediction equations from the basic, extended, and maximal models in the overall sample. Additional information about the linear regression models predicting absolute CRF can be found in Supplementary Table S4. Moreover, the Bland–Altman plots can also be found in Supplementary Figure S1, which did not show a systematic bias proportional to the measured value for eCRF basic (mean bias − 72 ml and 95% limits 468–612 ml) (Figure S1A), eCRF extended (mean bias − 71 ml and 95% limits 452–595 ml) (Figure S1B), and eCRF full (mean bias − 12 ml and 95% limits 378–401 ml) (Figure S1C).

**Table 3:** Fifteen best prediction equations for CRF^*^

	Equations	Cp	*r*^2^
Basic			
eCRF basic 1	** − 1261.99 + 1.97 × 6-min walking test (m) + 1.12 × BMR bioimpedance (kcal·day**^**-1**^**) + 5.25 × Basal HR (bpm)**	**2.05**	**0.74**
eCRF basic 2	− 1335.76 + 2.08 × 6-min walking test (m) + 0.99 × BMR bioimpedance (kcal·day^-1^) + 5.12 × Basal HR (bpm) + 2.92 × weight (kg)	2.43	0.75
eCRF basic 3	− 1405.67 × 1.76 × 6-min walking test (m) + 1.17 × BMR bioimpedance (kcal·day^-1^) + 4.67 × Basal HR (bpm) + 20.32 × chair stand test (rep)	3.62	0.75
eCRF basic 4	− 1329.77 × 2.02 × 6-min walking test (m) + 1.10 × BMR bioimpedance (kcal·day^-1^) + 5.21 × Basal HR (bpm) + 0.84 × waist circumference (cm)	3.65	0.75
eCRF basic 5	− 1280.63 × 1.94 × 6-min walking test (m) + 1.05 × BMR bioimpedance (kcal·day^-1^) + 7.45 × Basal HR (bpm) + 18.26 × PA recommendations (1, yes meeting; 0, non meeting)	3.73	0.74
Extended			
eCRF extended 1	** − 1291.11 + 1.70 × 6-min walking test (m) + 1.05 × BMR bioimpedance (kcal·day**^**-1**^**) + 5.11 × Basal HR (bpm) + 121.85 × forced expiratory volume in 1 s (L)**	**3.72**	**0.75**
eCRF extended 2	− 1457.44 + 1.93 × 6-min walking test (m) + 0.769 × BMR bioimpedance (kcal·day^-1^) + 5.10 × Basal HR (bpm) + 128.89 × forced expiratory volume in 1 s (l) + 5.46 × weight (kg)	4.16	0.76
eCRF extended 3	− 1418.91 + 1.92 × 6-min walking test (m) + 0.79 × BMR bioimpedance (kcal·day-1) + 5.11 × Basal HR (bpm) + 128.39 × forced expiratory volume in 1 s (l) – 0.66 × waist circumference (cm)	4.68	0.77
eCRF extended 4	− 1466.97 + 1.94 × 6-min walking test (m) + 6.22 × Basal HR (bpm) + 119.55 × forced expiratory volume in 1 s (l) + 15.52 × weight (kg) + 7.31 × Handgrip test (kg)	4.97	0.76
eCRF extended 5	− 1832.30 + 2.62 × 6-min walking test (m) + 6.18 × Basal HR (bpm) + 162.03 × forced expiratory volume in 1 s (l) + 19.42 × weight (kg)	5.03	0.78
Maximal			
eCRF maximal 1	** − 1297.61 + 1.10 × BMR bioimpedance (kcal·day**^**-1**^**) + 97.45 × forced expiratory volume in 1 s (L) + 5.95 × maximum HR in CPET (bpm) + 37.99 time to exhaustion in CPET (min)**	** − 2.29**	**0.87**
eCRF maximal 2	− 1374.95 + 1.09 × BMR bioimpedance (kcal·day^-1^) + 2.18 × Basal HR (bpm) + 96.71 × forced expiratory volume in 1 s (L) + 5.53 × maximum HR in CPET (bpm) + 39.94 × time to exhaustion in CPET (min)	− 1.76	0.86
eCRF maximal 3	− 1349.02 + 0.94 × BMR bioimpedance (kcal·day^-1^) + 103.23 × forced expiratory volume in 1 s (L) + 5.83 × maximum HR in CPET (bpm) + 40.31 × time to exhaustion in CPET (min) + 3.70 × weight (kg)	− 1.35	0.87
eCRF maximal 4	− 1423.14 + 0.93 × BMR bioimpedance (kcal·day^-1^) + 2.12 × Basal HR (bpm) + 102.41 × forced expiratory volume in 1 s (l) + 5.44 × maximum HR in CPET (bpm) + 42.15 × time to exhaustion in CPET (min) + 3.64 × weight (kg)	− 1.20	0.87
eCRF maximal 5	− 1481.40 + 0.89 × BMR bioimpedance (kcal·day^-1^) + 3.48 × Basal HR (bpm) + 99.38 × forced expiratory volume in 1 s (l) + 5.40 × maximum HR in CPET (bpm) + 41.47 × time to exhaustion in CPET (min) + 4.19 × weight (kg) + 16.40 × PA recommendations (1, yes meeting; 0, non meeting)	-0.86	0.87

Data in bold shows the best model for each condition (Basic, Extended and Maximal)

^*^Prediction equations used absolute peak oxygen consumption (ml/min) as indicator of CRF

*BMR*, basal metabolic rate; *Cp*, Mallow’s Cp; *HR*, heart rate; *PA*, physical activity; *r*^*2*^, adjusted *R*-squared

In the basic model, the first five equations were significant (all *p* < 0.001) and provided high prediction levels (*r*^2^ range from 0.74 to 0.75 and Mallow’s Cp range from 2.05 to 3.73). The best equation obtained (eCRF basic 1) included the 6-min walking test, eBMR, and basal HR explaining 74% of the variability of the absolute CRF level.

In the extended model, when spirometry parameters were added to the previous model, the prediction values were slightly increased. Thus, the best five prediction equations reported significant predictions of absolute CRF levels ranging from 0.75 to 0.78 (all *p* < 0.001, Mallow’s Cp from 3.72 to 5.03). The best equation (eCRF extended 1) included eBMR, 6-min walking test, basal HR, and FEV_1_, explaining 75% of the variability of the absolute CRF level.

Finally, when variables from the CPET (Maximum HR, time to exhaustion, and RPE max) were additionally added to the maximal model, the prediction value of the five best equations increased with *r*^2^ values ranging from 0.86 to 0.87 (all *p* < 0.001, Mallow’s Cp from − 2.29 to − 0.86). The best equation (eCRF maximal 1) explained 87% of the variability of the absolute CRF level.

Sensitivity analyses were performed only for those participants achieving maximal criteria in CPET, and the prediction value did not change (Supplementary Table S5, *n* = 68). Additionally, when the analyses were repeated using relative CRF instead of absolute CRF, the *r*^2^ values were similar including more predictors (data not shown).

Additionally, Supplementary Table S6 shows that delta (∆) in the best prediction equation models for eCRF developed in the present work was similar to objectively measured CRF by indirect calorimetry (~ ± 1 ml/kg/min).

### Associations of CRF and eCRF with cognitive performance

The multiple linear regression analyses reported in Table 4 show that both objectively measured CRF and eCRF levels were associated with better performance in BNT, COWAT, TMT, Digit Span Task (backward and forward), and the *z*-score after controlling for all the relevant confounders (sex, age, and education level) (all *p* < 0.050). When the analyses were repeated only for those achieving maximal criteria in CPET, the results did not change for either CRF or eCRF (data not shown).

**Table 4:** Associations of CRF^a^ and eCRF^b^ with cognitive performance in the overall sample

	Mini-Mental State Examination		Boston Naming Test		Clock Drawing Test		Rey Auditory Verbal Learning Test		Trail Making Test		Controlled Oral World Association		Stroop Test		WAIS Digit Span backward		WAIS Digit Span forward		*z*-score*	
	*β*	*p *value	*β*	*p *value	β	*p *value	*β*	*p *value	*β*	*p *value	*β*	*p *value	*β*	*p *value	*β*	*p *value	*β*	*p *value	*β*	*p *value
CRF^a^																				
Model 1	0.056	0.599	0.348	** < 0.001**	0.046	0.663	− 0.120	0.255	− 0.196	0.064	0.276	**0.008**	− 0.028	0.793	0.180	0.168	0.076	0.609	0.275	**0.023**
Model 2	− 0.072	0.211	0.026	** < 0.001**	− 0.158	0.367	− 0.030	0.061	− 0.240	** < 0.001**	0.165	** < 0.001**	0.070	0.364	0.093	**0.002**	0.019	**0.044**	0.220	** < 0.001**
eCRF^b^ basic																				
Model 1	0.104	0.322	0.453	** < 0.001**	0.157	0.134	− 0.191	0.069	− 0.122	0.253	0.266	**0.010**	0.070	0.514	0.180	0.168	0.067	0.610	0.340	**0.004**
Model 2	0.063	0.219	0.258	** < 0.001**	0.073	0.461	− 0.160	0.048	− 0.116	**0.002**	0.150	** < 0.001**	0.389	0.122	0.323	** < 0.001**	0.034	**0.044**	0.238	** < 0.001**
eCRF^b^ extended																				
Model 1	0.106	0.320	0.470	** < 0.001**	0.137	0.199	− 0.165	0.120	− 0.153	0.154	0.300	**0.004**	0.068	0.531	0.180	0.168	0.067	0.609	0.345	**0.004**
Model 2	− 0.011	0.237	0.190	** < 0.001**	− 0.066	0.470	− 0.115	0.068	− 0.124	**0.002**	0.133	** < 0.001**	0.410	0.133	0.243	**0.003**	0.033	**0.040**	0.255	** < 0.001**
eCRF^b^ maximal																				
Model 1	0.063	0.554	0.382	** < 0.001**	0.104	0.329	− 0.115	0.280	− 0.212	**0.048**	0.308	**0.003**	0.041	0.707	0.180	0.168	0.067	0.610	0.301	**0.014**
Model 2	− 0.074	0.224	0.018	** < 0.001**	− 0.096	0.447	0.035	0.075	− 0.283	** < 0.001**	0.197	** < 0.001**	0.231	0.257	0.104	**0.004**	0.021	**0.041**	0.168	** < 0.001**

Statistically significant values are shown in bold

*β*, standardized coefficient; *CRF*, cardiorespiratory fitness; *WAIS*, Wechsler Adult Intelligence Scale; *Model 1*, unadjusted model; *Model 2*, analyses adjusted for sex, age and education years

^a^CRF as indicator of absolute VO_2peak_ obtained by indirect calorimetry

^b^eCRF have been obtained of the best equation from each model (Table 3. 1, basic equation; 2, extended equation; 3, maximal equation)

^*^Mean value of the standardization of the scores of the nine neuropsychological tests shown

## Discussion

The main findings of this study were as follows: (i) to provide new equations with good predictive value for eCRF (74%-87%), specifically developed for older adults and using different scenarios of complexity (laboratory-based test and field-based test) and/or equipment requirements, and (ii) higher eCRF, even using its simplest equation, was associated with better performance on several cognitive dimensions (i.e., language, cognitive flexibility, fluency, attention, and working memory), similar to using objectively measured CRF.

### Prediction equations for cardiorespiratory fitness

The present study developed new equations specifically for older adults using both non-maximal exercise information (the basic and extended equations) and adding several complementary variables from CPET but without gas exchange (maximal equation). All the equations achieved high prediction values (74–87% of variance explained) above the average of equations previously reported and based on larger sample sizes and different population age groups [15, 51, 52]. Particularly, our *basic equation* for eCRF achieved 74% of explained variance, which has previously been achieved in only 14.8% of the equations (4 of the 27) previously published in the literature for eCRF [51]. Indeed, most of the equations provided values lower than our *non-maximal basic equation* (mean 61%, range 43–70%) [51].

The number and type of variables included in the calculation of eCRF are also relevant as they could affect the feasibility of these equations at different settings (clinical, epidemiology, etc.). Our *non-maximal basic equation* only used 3 variables such as the 6-min walking test, eBMR by bioimpedance, and basal HR. However, the 4 previously published equations reporting similar predictive values included 4 (fat percentage, physical activity, age, and sex) [53], 5 (age, sex, physical activity, height, and weight [54] or age, sex, BMI, current smoking status, and physical activity [55]), and 6 variables (age, sex, physical activity, fat percentage, current smoking status, and respiratory exchange ratio) [56]. Moreover, these equations were not specific for older people.

In this line, the equation proposed by Jurcal et al. [17] with a predictive value of 65% includes 5 variables (sex, age, BMI, resting HR, and physical activity) in a population of 20 to 70 years; however, this equation has some limitations to be applied in our population such as the characteristics of the original sample being very heterogeneous as indicated by the original study in its limitations. At a later stage, Jackson et al. [15] reported four equations by sex requiring 6 measurements to calculate eCRF (age, body fat percentage or BMI, waist circumference, basal HR, smoking, and physical activity level), and their predictive values ranged from 56 to 60%. Similarly, Nes et al. [16] developed two equations with a 56% and 61% of the variance explained and included five predictors to obtain eCRF (sex, age, waist circumference, resting HR, and physical activity). In short, the previous works used a higher number of predictors, and these were not specific equations for older people.

The observed differences in the predictive values found across studies could be due to the methodological variability identified. The different age ranges and sample sizes of age groups could be plausible reasons for the variability observed in the accuracy of non-maximal exercise prediction equations in previous studies [51]. However, despite such variability, most of these equations for eCRF have reported associations with the risk of hospitalizations [5], the incidence of strokes [57], or mortality [58, 59]. Therefore, it is noteworthy that this study obtained a simple (by using 3 variables) and age-specific equation with non-maximal exercise variables with a high accuracy. The present non-maximal exercise basic equation may be easily implemented as part of clinical evaluations at nursing homes and/or epidemiological studies to avoid physical and health risks associated with CPET in older adults [5], including the 6-min walking test, which is a non-maximal exercise and easily administered test currently used in daily clinical practice, and can be widely performed without the need of sophisticated equipment [60]. Moreover, the present study has shown that the 6-min test per se explains only 22% of the VO_2peak_ variability (Table 2). Moreover, we have calculated previous equations to predict VO_2peak_ using the 6-min test [61–64], obtaining predictive values lower than the predictive values of the new set of eCRF equations developed in this study (data not shown).

### Associations of CRF and eCRF with cognitive performance

Another main finding from our study was the association of the eCRF with key cognitive domains such as language, fluency, cognitive flexibility, attention, and working memory, independently of confounders. In line with our results, the study of McAuley et al. [65] reported associations of both objective CRF and eCRF with processing speed and memory. Yet, most research has been based only on objectively measured CRF [66] when analyzing its role with cognitive performance. Briefly, a recent systematic review showed a positive association between objective CRF and memory in older adults [66], and a prospective cohort study supported the association between higher CRF and greater fluency in noninstitutionalized older adults [67]. Moreover, Verstynen et al. [68] also showed that CRF was associated with a measure of cognitive flexibility.

Our findings did not show associations of eCRF levels with cognitive status, inhibition, and processing speed in accordance with previous studies [69, 70]. Although, several other studies reported significant associations of CRF with these cognitive domains [65, 71]. Differences in characteristics and size of the sample and methodological procedures could explain the disagreement among findings. For example, Boots et al. 2015 [23] have shown that their equation for eCRF was associated with cognitive function; however, this work included people between 20 and 70 years old; therefore, the equation was not specific for older people, and the *r*^2^ (~ 60%) was lower than either other equations available in the literature or the equations provided in our study. Furthermore, although Boots et al. 2015 [23] showed good accuracy for this equation to be associated with objectively measured CRF and cognitive function, the authors indicated as a potential limitation that they modified the original equation of Jurca et al. 2005 [17], being possible that this modification affected the results of the study.

Altogether, these findings indicate that eCRF is a useful approach for monitoring aerobic capacity when other methods are not available. Indeed, Tari et al. [4] found that a change in eCRF is an independent risk factor for dementia incidence and mortality. Hence, improving CRF could potentially be a key preventive strategy to avoid complex multi-morbidity [4] and specifically, dementia, the main non-communicable disorder in older adults [2]. Literature suggests that brain-derived neurotrophic factor (BDNF) is a mediator of the relationship between CRF and cognitive performance [72, 73] and that this circulating biomolecule, induced by exercise, may cross the blood–brain barrier and be important in protecting against neurodegenerative disorders, such as dementia [74]. Therefore, CRF is protective for brain function [66, 75] and might be related to better cognition and reduced risk of Alzheimer’s disease [4, 66]. For that reason, it is relevant to keep the aging population fit for longer because it could have huge positive public health and economic implications [8]. Therefore, physical activity recommendations should focus on activities with intensities that are proven to be effective in enhancing CRF [8].

This study has some limitations that should be taken into consideration when interpreting its results. Firstly, the age range included only older adults between 65 and 75 years; thus, this homogeneity in age limits the generalization to other ages among older adults; however, the present work has provided a new specific set of eCRF equations for older people with high predictive values. Moreover, the cross-sectional nature of the analyses does not allow to determine the causality between fitness and cognitive performance. However, we have proposed a large number of equations with a good prediction of CRF level, despite the limited sample size used. Other studies with larger samples have also generated equations to predict CRF in adults, the elderly, or both [15, 16, 51, 52]; however, these equations are not specific for older people, and there is a high variability between the equations available to estimate CRF, thus reducing their potential clinical utility.

## Conclusions

A new specific set of eCRF equations for older people have been developed with predictive values ranging from 74 to 87% that could be used based on needs, availability of equipment, resources, or measurement context (i.e., clinical setting or nursing home). Moreover, the eCRF is positively associated, similarly with objectively measured CRF, with performance on language, fluency, cognitive flexibility, attention, and working memory, independently of sex, age, and education level. This suggests that the new eCRF equations are useful as a proxy of CRF but also relate to cognitive performance. Thus, increasing CRF could be a protective factor against the deterioration of cognitive function associated with aging in older adults.

## Supplementary information

Below is the link to the electronic supplementary material.

Supplementary file1 (DOCX 311 KB)
