# Supplementary material for: A new set of estimated cardiorespiratory fitness equations are associated with cognitive performance in older adults
Source: GeroScience. 2023 Jan 19;45(3):1649–66. doi: 10.1007/s11357-022-00718-w (PMC10400484; doi:10.1007/s11357-022-00718-w)
Supplement: Supplementary file 1 — Supplementary file1 (DOCX 311 KB) [file 11357_2022_718_MOESM1_ESM.docx]

**SUPPLEMENTARY TABLES:**

**Table S1**. Additional descriptive characteristics of the study participants.

| **Variable** |  | **Total sample** n=92 | | | **Male** n=51 | | | **Female** n=41 | | | **p-value** |
| --- | --- | --- | --- | --- | --- | --- | --- | --- | --- | --- | --- |
|  |  |  |  |  |  |  |  |  |  |  |  |
| **Physical characteristics** |  |  |  |  |  |  |  |  |  |  |  |
| Body mass index status (%) (NW/OW/Ob) |  | 18 / 51 / 31 | | | 17 / 46 / 37 | | | 20 / 56 / 24 | | | 0.409 |
| Fat mass (%) |  | 31.0 | ± | 7.9 | 26.1 | ± | 5.8 | 37.3 | ± | 5.4 | **<0.001** |
| Fat mass (kg) |  | 23.3 | ± | 7.9 | 21.6 | ± | 8.0 | 25.4 | ± | 7.3 | **0.017** |
| Fat Free mass (%) |  | 69.0 | ± | 7.9 | 73.9 | ± | 5.8 | 62.7 | ± | 5.4 | **<0.001** |
| Fat Free mass (kg) |  | 51.2 | ± | 10.3 | 58.9 | ± | 6.9 | 41.6 | ± | 3.4 | **<0.001** |
| Fat mass index (kg·m^-2^) |  | 9.1 | ± | 3.4 | 7.8 | ± | 2.8 | 10.8 | ± | 3.3 | **<0.001** |
| Fat free mass index (kg·m^-2^) |  | 19.6 | ± | 2.4 | 21.1 | ± | 1.8 | 17.7 | ± | 1.7 | **<0.001** |
| Waist circumference (cm) |  | 99.2 | ± | 11.6 | 103.2 | ± | 10.7 | 94.2 | ± | 10.7 | **<0.001** |
| Hip circumference (cm) |  | 104.4 | ± | 9.3 | 103.3 | ± | 9.1 | 105.9 | ± | 9.3 | 0.177 |
| Waist to hip ratio (ratio) |  | 0.95 | ± | 0.08 | 0.99 | ± | 0.05 | 0.89 | ± | 0.06 | **<0.001** |
| **Resting cardiovascular parameters** |  |  |  |  |  |  |  |  |  |  |  |
| Mean blood pressure (mmHg) |  | 98.5 | ± | 10.8 | 99.5 | ± | 10.1 | 97.2 | ± | 11.7 | 0.330 |
| Systolic blood pressure (mmHg) |  | 136.8 | ± | 18.3 | 138.2 | ± | 17.2 | 135.1 | ± | 19.6 | 0.419 |
| Diastolic blood pressure (mmHg) |  | 79.4 | ± | 9.1 | 80.2 | ± | 8.6 | 78.3 | ± | 9.6 | 0.327 |
| HR basal (bpm) |  | 68.4 | ± | 10.8 | 68.7 | ± | 12.0 | 67.9 | ± | 9.1 | 0.738 |
| Double product (mmHg·bpm) |  | 9336 | ± | 1795 | 9484 | ± | 1933 | 9147 | ± | 1605 | 0.372 |
| Systolic/diastolic blood pressure (ratio) |  | 1.73 | ± | 0.19 | 1.73 | ± | 0.18 | 1.73 | ± | 0.19 | 0.944 |
| **Resting metabolic parameters** |  |  |  |  |  |  |  |  |  |  |  |
| Respiratory exchange ratio (ratio) |  | 0.77 | ± | 0.06 | 0.78 | ± | 0.07 | 0.76 | ± | 0.05 | 0.202 |
| **Spirometry*** |  |  |  |  |  |  |  |  |  |  |  |
| PEF (L·s^-1^) |  | 4.86 | ± | 1.80 | 5.50 | ± | 1.91 | 4.08 | ± | 1.29 | **<0.001** |
| **Physical fitness measures** |  |  |  |  |  |  |  |  |  |  |  |
| *Cardiopulmonary exercise test* |  |  |  |  |  |  |  |  |  |  |  |
| Relative cardiorespiratory fitness  (ml·kg^-1^FFM·min^-1^) |  | 36.1 | ± | 6.2 | 36.7 | ± | 6.0 | 35.4 | ± | 6.4 | **<0.001** |
| Metabolic equivalents measured (METs) |  | 7.1 | ± | 1.5 | 7.8 | ± | 1.4 | 6.4 | ± | 1.2 | **<0.001** |
| Cardiorespiratory fitness estimated (METs) |  | 7.9 | ± | 1.7 | 8.8 | ± | 1.7 | 7.0 | ± | 1.1 | **<0.001** |
| Maximum respiratory exchange ratio (ratio) |  | 1.10 | ± | 0.12 | 1.14 | ± | 0.12 | 1.04 | ± | 0.10 | **<0.001** |
| Maximum heart rate (bpm) |  | 139.9 | ± | 19.5 | 141.8 | ± | 21.5 | 137.6 | ± | 16.6 | 0.302 |
| Theoretical maximum HR (bpm) | | 159.7 | ± | 2.1 | 159.7 | ± | 2.1 | 159.9 | ± | 2.1 | 0.675 |

Values are presented as mean ± standard deviation or percentages. T-test and Chi square statistics was applied. Statistically significant differences between sexes are highlighted in bold. HR means heart rate; FFM, fat free mass; FVC, forced vital capacity; NW, normalweight; METs, metabolic equivalents; Ob, obesity; OW, overweight; PEF, peak expiratory flow. *Subsample of 90 participants in all cases.

**Table S2.** Summary of previous eCRF equations characteristics from the original studies and the results of applying to the current population data of older adults.

|  |  | **Original Studies characteristics** | | **Results after applying to a new set of date from older adults** | | | |
| --- | --- | --- | --- | --- | --- | --- | --- |
| **Authors** | **Equation** | **R^2^ in the original study^a^** | **Number of predictors used** | **R^2^ in the current study^b^** | **Estimated VO_2peak_**  **(ml kg^-1^ min^-1^)^c^** | ∆**^d^** | **p value^e^** |
| Riddle et al 1980 †† | Men: (60−0.55 (age))·((4.13 (height [cm])/2.54−135))/2.2 |  | 3 | 0.22 | 17.31 | 9.81 | **<0.001** |
|  | Women: (48−0.37 (age))·((3.55 (height [cm])/2.54−106))/2.2 |  | 3 | 0.22 | 16.92 | 5.39 | **<0.001** |
| Wasserman et al 1994 | Men: PW=0.79 (height [cm])−60.7If PW=measured weight: [(50.72−0.372 (age))·(weight [kg])]·1.11If PW>measured weight: [(PW+(weight [kg])/2) (50.72−0.372 (age))]·1.11If PW<measured weight: [(50.72−0.372 (age)) (weight [kg])+6 ((weight [kg])−PW)] 1.11 |  | 4 |  |  |  |  |
|  | Women: PW=0.65 (height [cm])−42.8If PW=measured weight: [((weight [kg])+43)(22.78−0.17 (age)] 1.11If PW>measured weight: [((PW+(weight[kg])+86)/2)(22.78 −0.17 (age)] 1.11If PW<measured weight: [(PW+43)(22.78−0.17 (age))+6 (weight [kg])−PW)] 1.11 |  | 4 |  |  |  |  |
| Jang et al 2012 (model 1) | 50.543−0.069 (age)+13.525 (sex; men=1, women=0)−0.403 (BMI)−1.530 (CSS**) | 0.67 | 4 | 0.40 | 40.71 | -15.72 | **<0.001** |
| Baynard et al 2016 (BMI) | 77.96−10.35 (sex; men=0, women=1)−0.92 (BMI)−0.32 (age) | 0.57 | 3 | 0.37 | 23.77 | 1.22 | **<0.001** |
| Baynard et al 2016 (WC) | 88.35−14.79 (sex; men=0, women=1)−0.40 (WC)−0.27 (age) | 0.60 | 3 | 0.42 | 21.87 | 3.12 | **<0.001** |
| Myers et al 2017 | 79.9−0.39 (age)−13.7 (sex; men=0, women=1)−0.127 (weight [lbs]) | 0.62 | 3 | 0.37 | 21.60 | 3.39 | **<0.001** |
| de Souza et al 2018 | 45.2−0.35 (age)−10.9 (sex; men=1, women=2)−0.15 (weight [lbs])+0.68 (height [in])−0.46 | 0.62 | 4 | 0.38 | 21.75 | 3.24 | **<0.001** |
| Jackson et al 1990 (BMI) | 56.363+1.921 (PA*)−0.381 (age)−0.754 (BMI)+10.987 (sex; men=1, women=0) | 0.61 | 4 | 0.35 | 15.06 | 9.93 | **<0.001** |
| Jackson et al 1990 (%fat) | 50.513+1.589 (PA*)−0.289 (age)−0.552 (%fat)+5.863 (sex; men=1, women=0) | 0.66 | 4 | 0.37 | 17.18 | 7.81 | **<0.001** |
| Heil et al 1995 | 36.580−0.541 (%fat)+1.921 (PA*)+0.558 (age)−7.81E- 3 (age^2^)+3.706 (sex; men=1, women=0) | 0.74 | 4 | 0.37 | 21.62 | 3.37 | **<0.001** |
| Whaley et al 1995 (BMI) | 64.62−0.339 (age)+9.006 (sex; men=1, women=0)+2.069 (PA†)−0.601 (BMI)−0.143 (RHR)−0.409 (CSS) | 0.70 | 6 | 0.40 | 19.45 | 5.54 | **<0.001** |
| Whaley et al 1995 (%fat) | 61.66−0.328 (age)+5.45 (sex; men=1, women=0)+1.832 (PA†)−0.436 (% fat)−0.143 (RHR)−0.446 (CSS) | 0.73 | 6 | 0.39 | 18.34 | 6.65 | **<0.001** |
| Matthews et al 1999 | 34.142+0.133 (age)−0.005 (age2)+11.403 (sex; men=1, women=0)+1.463 (PA*)+9.170 (height [m])−0.254 (weight [kg]) | 0.74 | 5 | 0.35 | 20.18 | 4.81 | **<0.001** |
| Jurca et al 2005 (ACLS) | 18.81+2.49 (sex; men=1, women=0)−0.08 (age)−0.17 (BMI)−0.05 (RHR)+0.81 (PA1‡) +1.17 ( PA 2‡) +2.16 ( PA 3‡)+3.05 (PA4‡) | 0.60 | 5 | 0.40 | 10.71 | 14.28 | **<0.001** |
| Jurca et al 2005 (ADNFS) | 21.41+2.78 (sex; men=1, women=0)−0.11 (age)−0.17 (BMI)−0.05 (RHR)+0.35 (PA1‡)+0.29 (PA2‡)+0.64 (PA3‡) +1. 21 ( PA 4‡) | 0.58 | 5 | 0.40 | 8.514 | 16.476 | **<0.001** |
| Jurca et al 2005 (NASA) | 18.07+2.77 (sex; men=1, women=0)−0.10 (age)−0.1 BMI)−0.03 (RHR)+0.32 (PA1‡) +1. 0 6 ( PA 2‡) +1.76 ( PA 3‡)+3.03 (PA4‡ | 0.65 | 5 | 0.40 | 11.76 | 13.23 | **<0.001** |
| Wier et al 2006 (BMI) | 57.402−0.372 (age)+8.596 (sex; men=1, women=0)+1.396 (PA§)−0.683 (BMI) | 0.62 | 4 | 0.35 | 22.41 | 2.58 | **<0.001** |
| Wier et al 2006 (%fat) | 51.936−0.308 (age)+4.065 (sex; men=1, women=0)+1.217 (PA§)−0.483 (%fat) | 0.65 | 4 | 0.37 | 18.27 | 6.72 | **<0.001** |
| Wier et al 2006 (WC) | 59.416−0.327 (age)+11.488 (sex; men=1, women=0)+1.297 (PA§)−0.266 (WC) | 0.64 | 4 | 0.38 | 23.80 | 1.19 | **<0.001** |
| Nes et al 2011 | Men: 100.27−0.296 (age)+0.226 (PA¶)−0.369 (WC)−0.155 (RHR) | 0.61 | 5 | 0.43 | 29.54 | -2.42 | **<0.001** |
|  | Women: 74.736−0.247 (age)+0.198 (PA¶)−0.259 (WC)−0.114 (RHR) | 0.56 | 5 | 0.43 | 25.64 | -3.33 | **<0.001** |
| Cáceres et al 2012 (9 levels)^γ^ | Men: 47.189+0.394 (age)−0.282 (weight [kg])−4.289 (PA$)+0.231 (height [cm])−0.090 (RHR)−2.092 (dyslipidemia)−1.925 (hypertension) −2.901 (CSS**)−2.295 (diabetes mellitus) | 0.53 | 9 | 0.40 | 26.31 | 0.81 | **<0.001** |
|  | Women: 37.844+0.250 (age)−0.208 (weight [kg])−3.428 (PA$)+0.139 (height [cm])−0.053 (RHR)−1.327 (dyslipidemia)−1.009 (hypertension)−1.508 (CSS**) | 0.44 | 9 | 0.40 | 27.71 | -5.4 | **<0.001** |
| Cáceres et al 2012 (5 levels) | Men: 39.390+0.409 (age)−0.307 (weight [kg])−4.437 (PA$)+0.254 (height [cm])−3.081 (CSS**) | 0.51 | 6 | 0.35 | 20.54 | 6.58 | **<0.001** |
|  | Women: 31.733+0.244 (age)−0.219 (weight [kg])−3.598 (PA$) + 0 .151 (height [cm])−1.486 (CSS**) | 0.43 | 6 | 0.35 | 38.67 | -16.36 | **<0.001** |
| Jackson et al 2012 (BMI, 5 levels)‡‡ | Men: 20.8013+0.1610 (age)−0.0022 (age2)−0.2240 (BMI)−0.0334 (WC)−0.0375 (RHR)+0.2163 (PA1‡)+0.3447 (PA2‡)+0.7877 (PA3‡) +1.1961 (PA4‡)−0.4306 (CSS**) |  | 7 | 0.43 | 32.41 | 5.29 | **<0.001** |
|  | Women: 14.5493+0.1136 (age)−0.0016 (age2)−0.1500 (BMI)−0.0088 (WC)−0.0359 (RHR)+0.2091 (PA1‡)+0.2275 (PA2‡)+0.7021 (PA3‡) +1.0070 (PA4‡)−0.3005 (CSS**) |  | 7 | 0.43 | 33.36 | 11.04 | **<0.001** |
| Jackson et al 2012 (%fat, 5 levels)‡‡ | Men: 17.7357+0.1620 (age)−0.0021 (age2)−0.1057 (%fat)−0.0422 (WC)−0.0363 (RHR)+0.2153 (PA1‡)+0.3655 (PA2‡)+0.8092 (PA3‡) +1.1989 (PA4‡)−0.4378 (CSS*) |  | 7 | 0.43 | 33.705 | 6.58 | **<0.001** |
|  | Women: 13.4967+0.1200 (age)−0.0017 (age2)−0.0817 (%fat)−0.0140 (WC)−0.0342 (RHR)+0.2402 (PA‡)+0.2735 (PA2‡)+0.7432 (PA3‡) +1.0346 (PA4‡)−0.3207 (CSS**) |  | 7 | 0.43 | 30.38 | 8.07 | **<0.001** |
| Jackson et al 2012 (BMI, 2 levels)‡‡ | Men: 21.2870+0.1654 (age)−0.0023 (age2)−0.2318 (BMI)−0.0337 (WC)−0.0390 (RHR)+0.6351 (PA$)−0.4263 (CSS**) |  | 7 | 0.43 | 32.24 | 5.11 | **<0.001** |
|  | Women: (14.7873+0.1159 (age)−0.0017 (age2)−0.1534 (BMI)−0.0088 (WC)−0.0364 (RHR)+0.5987 (PA$)−0.2994 (CSS**) |  | 7 | 0.43 | 33.22 | 10.90 | **<0.001** |
| Jackson et al 2012 (%fat, 2 levels)20‡‡ | Men: 18.1395+0.1662 (age)−0.0022 (age2)−0.1077 (%fat)−0.0431 (WC)−0.0380 (RHR)+0.6429 (PA$)−0.4339 (CSS**) |  | 7 | 0.43 | 22.64 | -4.48 | **<0.001** |
|  | Women: 13.7415+0.1223 (age)−0.0018 (age2)−0.0819 (%fat)−0.0141 (WC)−0.0349 (RHR)+0.6061 (PA$)−0.3188 (CSS**) |  | 7 | 0.43 | 20.17 | -2.14 | **<0.001** |
| Jang et al 2012 (model 2) | 48.392−0.088 (age)+12.335 (sex; men=1, women=0)−0.386 (BMI)−0.621 (CSS**)+0.693 (PA§) | 0.73 | 5 | 0.35 | 37.86 | -12.87 | **<0.001** |

Table based and modified from Peterman et al. 2020 [14]. We add the last 4 columns to do some additional analysis with our sample.

%Fat indicates percentage body fat; ACLS, equation based on data from the Aerobics Center Longitudinal Study; ADNFS, equation based on data from the Allied Dunbar National Fitness Survey; BMI, body mass index; CRF, cardiorespiratory fitness; CSS, current smoking status; NASA, equation based on data from National Aeronautics and Space Administration/Johnson Space Center; PA, physical activity; PW, predicted weight; RHR, respiratory exchange ratio; VO_2max_, maximal oxygen consumption; WC, waist circumference (cm).PA with *NASA Physical Activity Scale, †BALL ST (Ball State Adult Fitness Longitudinal Lifestyle Study) PA scale, §NASA Physical Activity Status Scale, ¶Nord-Trøndelag Health Study (HUNT) questionnaire, or (no=0, yes=1); CSS **binary (no=0, yes=1).

^a^R^2^ in each study column shows r^2^ for each original paper.

^b^R^2^ to present study column shows r^2^ when we use the same predictors as these equations with our sample.

^c^Estimated VO_2peak_ (ml·kg^-1^·min^-1^) using each eCRF equation with our sample.

^d^∆ was calculated by: Objectively measured CRF by indirect calorimetry - each eCRF equation.

^e^P value shows the level of significance in each linear regression analyses when we apply the same predictors than the original study.

^γ^Dyslipidemia, hypertension, and diabetes mellitus were not taken into account.

††Prediction equation calculates absolute VO_2max_ but was converted to relative VO_2max_ for the study from Peterman, 2020.

‡‡Prediction equation calculates metabolic equivalents but was converted to relative VO_2max_ for the present study Peterman, 2020.

**Table S3.** Prediction of each previous equation model applied in our sample with objectively measured CRF of the present study.

| **Authors** | **Equation** | **R^2-a^** | **Estimated VO_2peak_**  **(ml kg^-1^ min^-1^)^b^** | **p value^c^** |
| --- | --- | --- | --- | --- |
| Riddle et al 1980 †† | Men: (60−0.55 (age))·((4.13 (height [cm])/2.54−135))/2.2 | 0.21 | 17.31 | **<0.001** |
|  | Women: (48−0.37 (age))·((3.55 (height [cm])/2.54−106))/2.2 | 0.18 | 16.92 | **<0.001** |
| Jang et al 2012 (model 1) | 50.543−0.069 (age)+13.525 (sex; men=1, women=0)−0.403 (BMI)−1.530 (CSS**) | 0.27 | 40.71 | **<0.001** |
| Baynard et al 2016 (BMI) | 77.96−10.35 (sex; men=0, women=1)−0.92 (BMI)−0.32 (age) | 0.25 | 23.77 | **<0.001** |
| Baynard et al 2016 (WC) | 88.35−14.79 (sex; men=0, women=1)−0.40 (WC)−0.27 (age) | 0.50 | 21.87 | **<0.001** |
| Myers et al 2017 | 79.9−0.39 (age)−13.7 (sex; men=0, women=1)−0.127 (weight [lbs]) | 0.10 | 21.60 | **0.005** |
| de Souza et al 2018 | 45.2−0.35 (age)−10.9 (sex; men=1, women=2)−0.15 (weight [lbs])+0.68 (height [in])−0.46 | 0.20 | 21.75 | **<0.001** |
| Jackson et al 1990 (BMI) | 56.363+1.921 (PA*)−0.381 (age)−0.754 (BMI)+10.987 (sex; men=1, women=0) | 0.36 | 15.06 | **<0.001** |
| Jackson et al 1990 (%fat) | 50.513+1.589 (PA*)−0.289 (age)−0.552 (%fat)+5.863 (sex; men=1, women=0) | 0.33 | 17.18 | **<0.001** |
| Heil et al 1995 | 36.580−0.541 (%fat)+1.921 (PA*)+0.558 (age)−7.81E- 3 (age^2^)+3.706 (sex; men=1, women=0) | 0.34 | 21.62 | **<0.001** |
| Whaley et al 1995 (BMI) | 64.62−0.339 (age)+9.006 (sex; men=1, women=0)+2.069 (PA†)−0.601 (BMI)−0.143 (RHR)−0.409 (CSS#) | 0.29 | 19.45 | **<0.001** |
| Whaley et al 1995 (%fat) | 61.66−0.328 (age)+5.45 (sex; men=1, women=0)+1.832 (PA†)−0.436 (% fat)−0.143 (RHR)−0.446 (CSS#) | 0.22 | 18.34 | **<0.001** |
| Matthews et al 1999 | 34.142+0.133 (age)−0.005 (age2)+11.403 (sex; men=1, women=0)+1.463 (PA*)+9.170 (height [m])−0.254 (weight [kg]) | 0.06 | 20.18 | **0.026** |
| Jurca et al 2005 (ACLS) | 18.81+2.49 (sex; men=1, women=0)−0.08 (age)−0.17 (BMI)−0.05 (RHR)+0.81 (PA1‡) +1.17 ( PA 2‡) +2.16 ( PA 3‡)+3.05 (PA4‡) | 0.28 | 10.71 | **<0.001** |
| Jurca et al 2005 (ADNFS) | 21.41+2.78 (sex; men=1, women=0)−0.11 (age)−0.17 (BMI)−0.05 (RHR)+0.35 (PA1‡)+0.29 (PA2‡)+0.64 (PA3‡) +1. 21 ( PA 4‡) | 0.28 | 8.514 | **<0.001** |
| Jurca et al 2005 (NASA) | 18.07+2.77 (sex; men=1, women=0)−0.10 (age)−0.1 BMI)−0.03 (RHR)+0.32 (PA1‡) +1. 0 6 ( PA 2‡) +1.76 ( PA 3‡)+3.03 (PA4‡ | 0.25 | 11.76 | **<0.001** |
| Wier et al 2006 (BMI) | 57.402−0.372 (age)+8.596 (sex; men=1, women=0)+1.396 (PA§)−0.683 (BMI) | 0.37 | 22.41 | **<0.001** |
| Wier et al 2006 (%fat) | 51.936−0.308 (age)+4.065 (sex; men=1, women=0)+1.217 (PA§)−0.483 (%fat) | 0.35 | 18.27 | **<0.001** |
| Wier et al 2006 (WC) | 59.416−0.327 (age)+11.488 (sex; men=1, women=0)+1.297 (PA§)−0.266 (WC) | 0.37 | 23.80 | **<0.001** |
| Nes et al 2011 | Men: 100.27−0.296 (age)+0.226 (PA¶)−0.369 (WC)−0.155 (RHR) | 0.06 | 29.54 | 0.090 |
|  | Women: 74.736−0.247 (age)+0.198 (PA¶)−0.259 (WC)−0.114 (RHR) | 0.15 | 25.64 | **0.011** |
| Cáceres et al 2012 (9 levels)^γ^ | Men: 47.189+0.394 (age)−0.282 (weight [kg])−4.289 (PA$)+0.231 (height [cm])−0.090 (RHR)−2.092 (dyslipidemia)−1.925 (hypertension) −2.901 (CSS**)−2.295 (diabetes mellitus) | 0.10 | 26.31 | **0.029** |
|  | Women: 37.844+0.250 (age)−0.208 (weight [kg])−3.428 (PA$)+0.139 (height [cm])−0.053 (RHR)−1.327 (dyslipidemia)−1.009 (hypertension)−1.508 (CSS**) | 0.01 | 27.71 | 0.723 |
| Cáceres et al 2012 (5 levels) | Men: 39.390+0.409 (age)−0.307 (weight [kg])−4.437 (PA$)+0.254 (height [cm])−3.081 (CSS**) | 0.15 | 20.54 | **0.006** |
|  | Women: 31.733+0.244 (age)−0.219 (weight [kg])−3.598 (PA$) + 0 .151 (height [cm])−1.486 (CSS**) | 0.06 | 38.67 | 0.133 |
| Jackson et al 2012 (BMI, 5 levels)‡‡ | Men: 20.8013+0.1610 (age)−0.0022 (age2)−0.2240 (BMI)−0.0334 (WC)−0.0375 (RHR)+0.2163 (PA1‡)+0.3447 (PA2‡)+0.7877 (PA3‡) +1.1961 (PA4‡)−0.4306 (CSS**) | 0.16 | 11.26 | **0.004** |
|  | Women: 14.5493+0.1136 (age)−0.0016 (age2)−0.1500 (BMI)−0.0088 (WC)−0.0359 (RHR)+0.2091 (PA1‡)+0.2275 (PA2‡)+0.7021 (PA3‡) +1.0070 (PA4‡)−0.3005 (CSS** | 0.19 | 11.53 | **0.005** |
| Jackson et al 2012 (%fat, 5 levels)‡‡ | Men: 17.7357+0.1620 (age)−0.0021 (age2)−0.1057 (%fat)−0.0422 (WC)−0.0363 (RHR)+0.2153 (PA1‡)+0.3655 (PA2‡)+0.8092 (PA3‡) +1.1989 (PA4‡)−0.4378 (CSS*) | 0.15 | 11.63 | **0.004** |
|  | Women: 13.4967+0.1200 (age)−0.0017 (age2)−0.0817 (%fat)−0.0140 (WC)−0.0342 (RHR)+0.2402 (PA‡)+0.2735 (PA2‡)+0.7432 (PA3‡) +1.0346 (PA4‡)−0.3207 (CSS**) | 0.18 | 10.68 | **0.006** |
| Jackson et al 2012 (BMI, 2 levels)‡‡ | Men: 21.2870+0.1654 (age)−0.0023 (age2)−0.2318 (BMI)−0.0337 (WC)−0.0390 (RHR)+0.6351 (PA$)−0.4263 (CSS**) | 0.16 | 11.21 | **0.004** |
|  | Women: (14.7873+0.1159 (age)−0.0017 (age2)−0.1534 (BMI)−0.0088 (WC)−0.0364 (RHR)+0.5987 (PA$)−0.2994 (CSS**) | 0.19 | 11.49 | **0.005** |
| Jackson et al 2012 (%fat, 2 levels)20‡‡ | Men: 18.1395+0.1662 (age)−0.0022 (age2)−0.1077 (%fat)−0.0431 (WC)−0.0380 (RHR)+0.6429 (PA$)−0.4339 (CSS**) | 0.19 | 8.47 | **0.005** |
|  | Women: 13.7415+0.1223 (age)−0.0018 (age2)−0.0819 (%fat)−0.0141 (WC)−0.0349 (RHR)+0.6061 (PA$)−0.3188 (CSS**) | 0.23 | 8.30 | **<0.001** |
| Jang et al 2012 (model 2) | 48.392−0.088 (age)+12.335 (sex; men=1, women=0)−0.386 (BMI)−0.621 (CSS**)+0.693 (PA§) | 0.29 | 37.86 | **<0.001** |

Table based and modified from Peterman et al. 2020 [14]. We add the last 3 columns to do some additional analysis with our sample.

%Fat indicates percentage body fat; ACLS, equation based on data from the Aerobics Center Longitudinal Study; ADNFS, equation based on data from the Allied Dunbar National Fitness Survey; BMI, body mass index; CRF, cardiorespiratory fitness; CSS, current smoking status; NASA, equation based on data from National Aeronautics and Space Administration/Johnson Space Center; PA, physical activity; PW, predicted weight; RHR, respiratory exchange ratio; VO_2max_, maximal oxygen consumption; WC, waist circumference (cm).PA with *NASA PA Scale, †BALL ST (Ball State Adult Fitness Longitudinal Lifestyle Study) PA scale, §NASA Physical Activity Status Scale, ¶Nord-Trøndelag Health Study (HUNT) questionnaire, or (no=0, yes=1); CSS (no=0, yes=1).

^a^R^2^ column shows r^2^ when we did the predictive regression models applying the equation of each original study using this study data of older adults.

^b^Estimated VO_2peak_ (ml·kg^-1^·min^-1^) using each eCRF equation with our sample.

^c^P value shows the level of significance when we apply the equation of each original study using this study data of older adults.

^γ^Dyslipidemia, hypertension, and diabetes mellitus were not taken into account.

††Prediction equation calculates absolute VO_2ma_x but was converted to relative VO_2max_ for the study from Peterman, 2020.

‡‡Prediction equation calculates metabolic equivalents but was converted to relative VO_2max_ for the present study Peterman, 2020 and when we did the analyses with our sample.

**Table S4.** Linear regression models predicting absolute CRF.

| **BASIC MODELS** | | | | | | | | | | |
| --- | --- | --- | --- | --- | --- | --- | --- | --- | --- | --- |
| **Coefficients** | **Model 1** | | **Model 2** | | **Model 3** | | **Model 4** | | **Model 5** | |
| R^2^ |  | 0.743 |  | 0.750 |  | 0.745 |  | 0.745 |  | 0.744 |
| R^2^ adjusted |  | 0.731 |  | 0.734 |  | 0.728 |  | 0.728 |  | 0.728 |
| Cp |  | 2.046 |  | 2.429 |  | 3.623 |  | 3.647 |  | 3.733 |
| AIC |  | 921.02 |  | 921.22 |  | 922.55 |  | 922.58 |  | 922.67 |
| BIC |  | 929.78 |  | 932.16 |  | 933.50 |  | 933.53 |  | 933.62 |
| p value for model |  | **<0.001** |  | **<0.001** |  | **<0.001** |  | **<0.001** |  | **<0.001** |
| **Variables** | **β** | ***p value*** | **β** | ***p value*** | **β** | ***p value*** | **β** | ***p value*** | **β** | ***p value*** |
| eBMR (kcal·day^-1^) | 0.715 | **<0.001** | 0.590 | **0.001** | 0.710 | **<0.001** | 0.753 | **<0.001** | 0.698 | **<0.001** |
| 6 min walking (m) | 0.351 | **<0.001** | 0.378 | **<0.001** | 0.330 | **<0.001** | 0.331 | **<0.001** | 0.288 | **<0.001** |
| HR basal (bpm) | 0.136 | **0.036** | 0.132 | **0.042** | 0.136 | **0.038** | 0.139 | **0.033** | 0.168 | **0.019** |
| Weight (kg) | -- | -- | 0.135 | 0.408 | -- | -- | -- | -- | -- | -- |
| Chair stand (rep) | -- | -- | -- | -- | 0.038 | 0.654 | -- | -- | -- | -- |
| WC (cm) | -- | -- | -- | -- | -- | -- | -0.051 | 0.620 | -- | -- |
| Meeting PAr, (yes, no) | -- | -- | -- | -- | -- | -- | -- | -- | 0.052 | 0.461 |
| **EXTENDED MODELS** | | | | | | | | | | |
| **Coefficients** | **Model 1** | | **Model 2** | | **Model 3** | | **Model 4** | | **Model 5** | |
| R^2^ |  | 0.750 |  | 0.757 |  | 0.771 |  | 0.762 |  | 0.778 |
| R^2^ adjusted |  | 0.733 |  | 0.736 |  | 0.743 |  | 0.737 |  | 0.747 |
| Cp |  | 3.715 |  | 4.160 |  | 4.682 |  | 4.973 |  | 5.025 |
| AIC |  | 908.24 |  | 908.49 |  | 908.41 |  | 909.13 |  | 908.38 |
| BIC |  | 919.11 |  | 921.54 |  | 925.81 |  | 924.35 |  | 927.95 |
| p value for model |  | **<0.001** |  | **<0.001** |  | **<0.001** |  | **<0.001** |  | **<0.001** |
| **Variables** | **β** | ***p value*** | **β** | ***p value*** | **β** | ***p value*** | **β** | ***p value*** | **β** | ***p value*** |
| eBMR (kcal·day^-1^) | 0.615 | **<0.001** | 0.645 | **0.001** | 0.627 | **<0.001** | 0.646 | **<0.001** | 0.642 | **<0.001** |
| 6 min walking (m) | 0.281 | **<0.001** | 0.246 | **<0.001** | 0.290 | **<0.001** | 0.280 | **<0.001** | 0.251 | **0.001** |
| HR basal (bpm) | 0.094 | 0.133 | 0.082 | 0.168 | 0.056 | 0.386 | -- | -- | 0.056 | 0.379 |
| Weight (kg) | -- | -- | -- | -- | -- | -- | -- | -- | -- | -- |
| Chair stand (rep) | -- | -- | 0.085 | 0.245 | -- | -- | -- | -- | 0.090 | 0.221 |
| FEV_1_ (L) | 0.174 | **0.009** | 0.126 | **0.045** | 0.153 | **0.018** | 0.152 | **0.019** | 0.118 | 0.063 |
| Handgrip (kg) | -- | -- | -- | -- | -- | -- | -- | -- | -- | -- |
| Smoking (yes, no) | -- | -- | -- | -- | 0.134 | **0.048** | 0.153 | **0.022** | 0.100 | 0.120 |
| **MAXIMAL MODELS** | | | | | | | | | | |
| **Coefficients** | **Model 1** | | **Model 2** | | **Model 3** | | **Model 4** | | **Model 5** | |
| R^2^ |  | 0.869 |  | 0.863 |  | 0.872 |  | 0.871 |  | 0.866 |
| R^2^ adjusted |  | 0.860 |  | 0.856 |  | 0.861 |  | 0.860 |  | 0.857 |
| Cp |  | -2.294 |  | -1.757 |  | -1.349 |  | -1.196 |  | -0.855 |
| AIC |  | 866.15 |  | 867.21 |  | 866.84 |  | 867.03 |  | 867.90 |
| BIC |  | 877.02 |  | 875.90 |  | 879.88 |  | 880.08 |  | 878.77 |
| p value for model |  | **<0.001** |  | **<0.001** |  | **<0.001** |  | **<0.001** |  | **<0.001** |
| **Variables** | **β** | ***p value*** | **β** | ***p value*** | **β** | ***p value*** | **β** | ***p value*** | **β** | ***p value*** |
| eBMR (kcal·day^-1^) | 0.649 | **<0.001** | 0.693 | **<0.001** | 0.678 | **<0.001** | 0.641 | **<0.001** | 0.708 | **<0.001** |
| HR basal (bpm) | -- | -- | -- | -- | -- | -- | 0.040 | 0.415 | -- | -- |
| Weight (kg) | -- | -- | -- | -- | -- | -- | -- | -- | -- | -- |
| Chair stand (rep) | -- | -- | -- | -- | 0.042 | 0.379 | -- | -- | 0.060 | 0.215 |
| FEV_1_ (L) | 0.139 | **0.005** | -- | -- | 0.106 | **0.019** | 0.138 | **0.006** | -- | -- |
| HR max (bpm) | 0.237 | **<0.001** | 0.229 | **<0.001** | 0.271 | **<0.001** | 0.221 | **<0.001** | 0.269 | **<0.001** |
| Time exhaustion (min) | 0.295 | **<0.001** | 0.346 | **<0.001** | 0.242 | **<0.001** | 0.310 | **<0.001** | 0.274 | **<0.001** |

AIC means Akaike information criterion; β, standardized coefficient; BIC, Bayesian information criterion; eBMR, bioimpedance estimated basal metabolic rate; Cp, Mallow's Cp; FEV_1_, forced expiratory volume in 1 second; HR, heart rate; PAr, physical activity recommendations; WC, waist circumference.

| **Table S5:** Fifteen best prediction equations for CRF of participants achieving maximal criteria during cardiopulmonary exercise test. | | | |
| --- | --- | --- | --- |
|  | **Equations** | **Cp** | **r^2^** |
| **Basic** |  |  |  |
| **eCRF**  **basic 1** | **-1309.98 + 1.83 x 6 minutes walking test (m) + 1.19 x BMR bioimpedance (kcal/day) + 6.86 x Basal HR (bpm)** | **0.01** | **0.77** |
| eCRF  basic 2 | -1404.95 + 1.97 x 6 minutes walking test (m) + 0.98 x BMR bioimpedance (kcal/day) + 6.66 x Basal HR (bpm) + 4.61 x weight (kg) | 1.46 | 0.77 |
| eCRF  basic 3 | -1338.86 x 1.74 x 6 minutes walking test (m) + 1.18 x BMR bioimpedance (kcal/day) + 6.89 x Basal HR (bpm) + 7.75 x chair stand test (rep) | 1.58 | 0.77 |
| eCRF  basic 4 | -1155.09 x 1.73 x 6 minutes walking test (m) + 1.25 x BMR bioimpedance (kcal/day) + 7.05 x Basal HR (bpm) – 2.06 x waist circumference (cm) | 1.72 | 0.77 |
| eCRF  basic 5 | -1373.49 x 1.70 x 6 minutes walking test (m) + 1.17 x BMR bioimpedance (kcal/day) + 9.03 x Basal HR (bpm) +51.70 x PA recommendations (1, yes; 0, non meeting) | 1.73 | 0.77 |
| **Extended** |  |  |  |
| **eCRF extended 1** | **-1229.31 + 1.47 x 6 minutes walking test (m) + 1.11 x BMR bioimpedance (kcal/day) + 6.02 x Basal HR (bpm) + 116.95 x forced expiratory volume in 1 second (L)** | **3.31** | **0.78** |
| eCRF extended 2 | -1444.93 + 1.75 x 6 minutes walking test (m) + 0.69 x BMR bioimpedance (kcal/day) + 5.92 x Basal HR (bpm) + 141.52 x forced expiratory volume in 1 second (L) + 8.69 x weight (kg) | 3.70 | 0.79 |
| eCRF extended 3 | -1117.47 + 1.63 x 6 minutes walking test (m) + 0.66 x BMR bioimpedance (kcal/day) + 6.21 x Basal HR (bpm) + 134.86 x forced expiratory volume in 1 second (L) -5.56 x waist circumference (cm) | 4.43 | 0.80 |
| eCRF extended 4 | -1432.66 + 1.70 x 6 minutes walking test (m) + 6.24 x Basal HR (bpm) + 170.12 x forced expiratory volume in 1 second (L) + 18.12 x weight (kg) + 4.65 x Handgrip test (kg) | 4.73 | 0.79 |
| eCRF extended 5 | -1683.32 + 2.15 x 6 minutes walking test (m) + 6.16 x Basal HR (bpm) + 192.85 x forced expiratory volume in 1 second (L) + 20.89 x weight (kg) | 4.81 | 0.78 |
| **Maximal** |  |  |  |
| **eCRF maximal 1** | **-1427.30 + 1.13 x BMR bioimpedance (kcal/day) + 120.83 x forced expiratory volume in 1 second (L) + 6.67 x Maximum HR in CPET (bpm) + 32.90 x Time to exhaustion in CPET (min)** | **2.50** | **0.87** |
| eCRF maximal 2 | -1487.16 + 1.87 x BMR bioimpedance (kcal/day) + 121.69 x forced expiratory volume in 1 second (L) + 6.23 x Maximum HR in CPET (bpm) + 34.50 x Time to exhaustion in CPET (min) | 2.77 | 0.88 |
| eCRF maximal 3 | -1522.16 + 0.80 x BMR bioimpedance (kcal/day) + 148.00 x forced expiratory volume in 1 second (L) + 6.45 x Maximum HR in CPET (bpm) + 37.21 x Time to exhaustion in CPET (min) + 6.88 x weight (kg) | 2.92 | 0.88 |
| eCRF maximal 4 | -1580.32 +1.83 x Basal HR (bpm) + 0.69 x BMR bioimpedance (kcal/day) + 156.18 x forced expiratory volume in 1 second (L) + 5.52 x Maximum HR in CPET (bpm) + 38.75 x Time to exhaustion in CPET (min) + 6.85 x weight (kg) | 3.16 | 0.88 |
| eCRF maximal 5 | -1682.24 + 4.21 x Basal HR (bpm) + 0.79 x BMR bioimpedance (kcal/day) + 148.73 x forced expiratory volume in 1 second (L) + 6.02 x Maximum HR in CPET (bpm) + 38.68 x Time to exhaustion in CPET (min) + 8.60 x weight (kg) + 70.04 x PA recommendations (1, yes meeting; 0, non meeting) | 4.10 | 0.89 |
| BMR means basal metabolic rate; Cp, Mallow's Cp; HR, heart rate; PA, physical activity; r^2^, adjusted R-squared. | | | |

**Table S6.** Summary of the best prediction equation models for eCRF developed in the present work.

| **Model** | **Equation** | **Number of predictors used** | **R^2^ in the current study^a^** | **Estimated VO_2peak_**  **(ml·kg^-1^·min^-1^)^b^** | ∆**^c^** | **p value^d^** |
| --- | --- | --- | --- | --- | --- | --- |
| eCRF^e^  basic | -1261.99 + 1.97 x 6 minutes walking test (m) + 1.12 x BMR bioimpedance (kcal/day) + 5.25 x Basal HR (bpm) | 3 | 0.74 | 25.99 | -1 | **<0.001** |
| eCRF^e^  extened | -1291.11 + 1.70 x 6 minutes walking test (m) + 1.05 x BMR bioimpedance (kcal/day) + 5.11 x Basal HR (bpm) + 121.85 x forced expiratory volume in 1 second (L) | 4 | 0.75 | 25.97 | -0.98 | **<0.001** |
| eCRF^e^  maximal | -1291.11 + 1.70 x 6 minutes walking test (m) + 1.05 x BMR bioimpedance (kcal/day) + 5.11 x Basal HR (bpm) + 121.85 x forced expiratory volume in 1 second (L) | 4 | 0.87 | 25.15 | -0.16 | **<0.001** |
| CRF | Objectively measured VO_2peak_ by indirect calorimetry. |  |  | 24.99  Men:27.12  Women:22.31 |  |  |

BMR, basal metabolic rate; CRF, cardiorespiratory fitness; HR, respiratory exchange ratio; VO_2max_, maximal oxygen consumption;

^a^R^2^ to present study column shows r^2^ when we use the same predictors as these equations with our sample.

^b^Estimated VO_2peak_ (ml kg^-1^ min^-1^) using each eCRF equation with our sample.

^c^∆ was calculated by: Objectively measured CRF by indirect calorimetry - each eCRF equation.

^d^P value shows the level of significance when we use the same predictors as these equations with our sample.

^e^eCRF have been obtained of the best equation from each model (1, Basic Equation; 2, Extended Equation; 3, Maximal Equation).

**SUPPLEMENTARY FIGURE:**


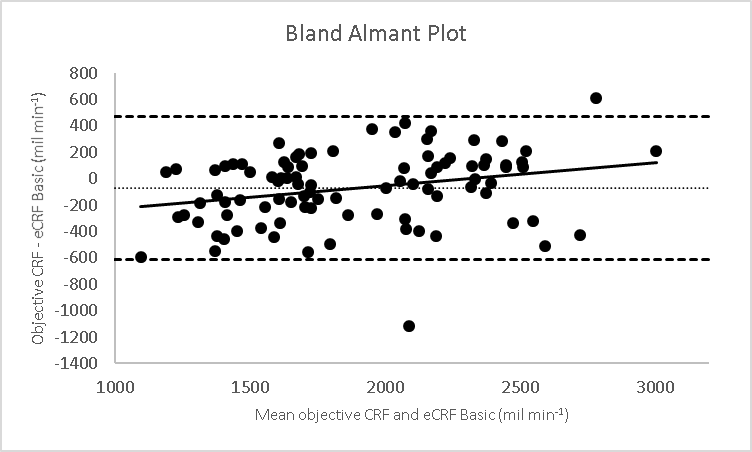

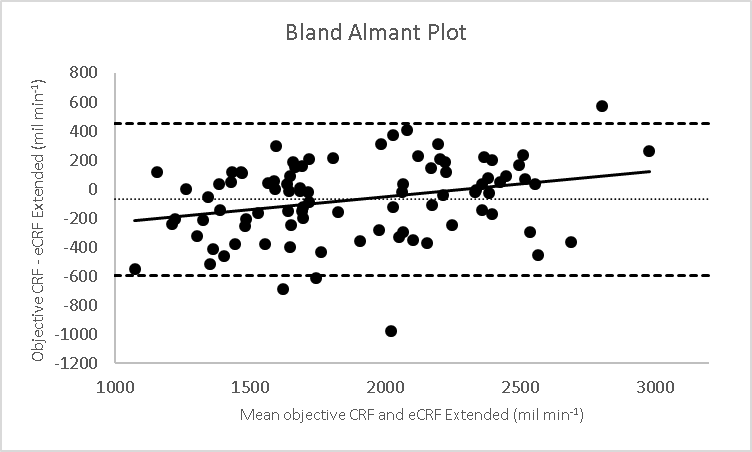

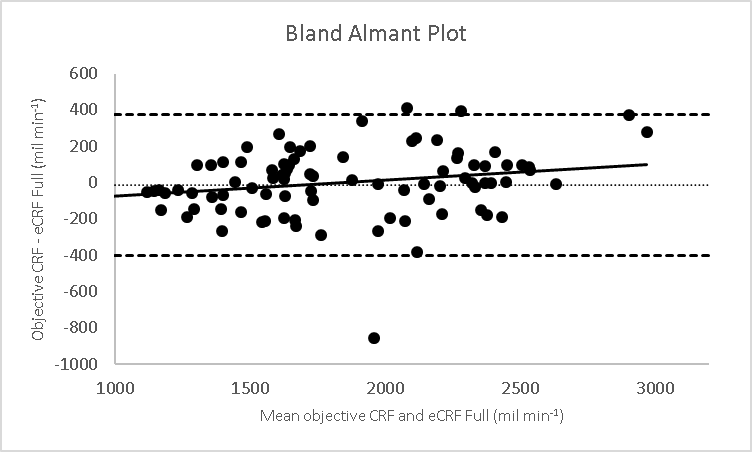


**A**

**B**

**C**


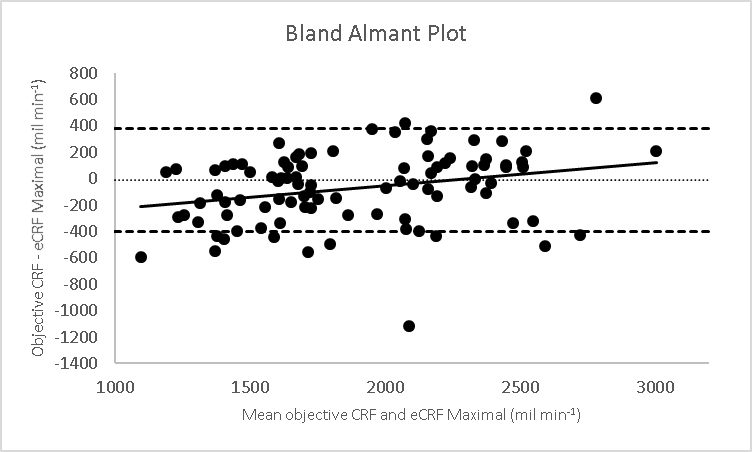

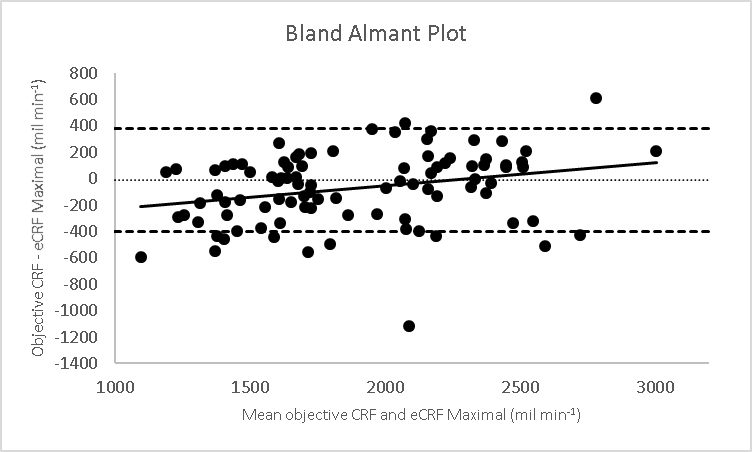


**Figure S1.** Bland‐Altman plots showing the mean of objectively measured CRF and (A) eCRF Basic (mean bias -72 ml and 95% limits 468–612 ml), (B) eCRF Extended (mean bias -71 ml and 95% limits 452–595 ml), and (C) eCRF Full (mean bias -12 ml and 95% limits 378–401 ml). CRF, Cardiorespiratory fitness; eCRF, estimated cardiorespiratory fitness. eCRF have been obtained of the best equation from eac h model (1, Basic Equation; 2, Extended Equation; 3, Maximal Equation).
